# Unveiling Potential Targeted Therapeutic Opportunities for Co-Overexpressed Targeting Protein for Xklp2 and Aurora-A Kinase in Lung Adenocarcinoma

**DOI:** 10.1007/s12033-023-00879-9

**Published:** 2023-09-28

**Authors:** Arnab Mukherjee, Preeti Harigovind Yadav, K. S. Mukunthan

**Affiliations:** 1 Department of Biotechnology, Manipal Institute of Technology, Manipal Academy of Higher Education, Manipal, Karnataka 576104 India; 2 Department of Bioinformatics, S.S. and L.S. Patkar College, Mumbai, India

**Keywords:** Lung adenocarcinoma, Biomarkers, Networks, Targeted therapeutics, Molecular docking

## Abstract

Lung adenocarcinoma (LUAD) is one of the most prevalent and leading causes of cancer deaths globally, with limited diagnostic and clinically significant therapeutic targets. Identifying the genes and processes involved in developing and progressing LUAD is crucial for developing effective targeted therapeutics and improving patient outcomes. Therefore, the study aimed to explore the RNA sequencing data of LUAD from The Cancer Genome Atlas (TCGA) and gene expression profile datasets involving GSE10072, GSE31210, and GSE32863 from the Gene Expression Omnibus (GEO) databases. The differential gene expression and the downstream analysis determined clinically significant biomarkers using a network-based approach. These therapeutic targets predominantly enriched the dysregulation of mitotic cell cycle regulation and revealed the co-overexpression of Aurora-A Kinase (AURKA) and Targeting Protein for Xklp2 (TPX2) with high survival risk in LUAD patients. The hydrophobic residues of the AURKA–TPX2 interaction were considered as the target site to block the autophosphorylation of AURKA during the mitotic cell cycle. The tyrosine kinase inhibitor (TKI) dacomitinib demonstrated the strong binding potential to hinder TPX2, shielding the AURKA destabilization. This in silico study lays the foundation for repurposing targeted therapeutic options to impede the Protein–Protein Interactions (PPIs) in LUAD progression and aid in future translational investigations.

**Supplementary Information:**

The online version contains supplementary material available at 10.1007/s12033-023-00879-9.

## Introduction

Lung cancer has emerged as the second most prevalent cancer and the leading cause of cancer-related death, posing a serious global health concern [1]. Tobacco smoking is the primary cause of lung cancer deaths worldwide, with men being more vulnerable than women [2]. Despite advances in research and therapy, LUAD remains a life-threatening malignancy that accounts for 40% of all lung cancer cases [3]. Despite advances in cancer treatment options, such as chemotherapy, immunotherapy, and non-invasive surgical resection, the 5-year overall survival (OS) rate for LUAD patients remains about 17.4% [4]. Therefore, it is imperative to understand the molecular mechanisms underlying the disease and identify key biomarkers to enable early detection and successful management of the disease. Technologies such as next-generation sequencing, microarrays, and proteomics have been instrumental in identifying biomarkers, but identifying key genes remains a challenge for developing targeted therapies to improve patient outcomes [5]. Network pharmacology addresses the multiple key factors and targets that interact to govern associated complex pathways [6].

Recently, the clinical results of targeted therapy at the molecular level for LUAD patients were promising. However, the obstacle of drug resistance continues to impede patients’ overall cure. Precision oncology has improved treatment results and quality of life compared to conventional chemotherapy since the emergence of genomic medicine [7]. Recent progress in understanding pathways, advancements in technologies for identifying genetic abnormalities, and the emergence of novel drugs to inhibit these pathways have enabled healthcare professionals to customize treatment approaches [8]. Several significant targetable pathways in lung adenocarcinoma have been discovered, including the Epidermal Growth Factor Receptor (EGFR), PI3K/AKT/mTOR, and RAS-MAPK pathways [9, 10]. Targeting EGFR mutations is the primary approach for treating LUAD [11]. Identifying these genetic alterations is crucial in clinical practice across the globe. Despite this, novel oncogenic drivers have recently emerged, resulting in clinically effective therapeutics that are either approved or in development [12]. Recent studies revealed that overexpression, amplification, and exon-skipping mutations in novel molecular targets such as Mesenchymal–Epithelial Transition factor (MET) and Neurotrophic Tyrosine Kinase (NTRK) are associated with aggressiveness, metastasis, vascular invasion, and drug resistance ultimately impacting the poor prognosis of LUAD population [13, 14]. Numerous drugs that target these pathways have been developed and demonstrated therapeutic effects. Some of these, such as the EGFR inhibitors erlotinib and gefitinib and the PI3K/AKT/mTOR inhibitor everolimus, have now been supplanted as the first-line treatment [15, 16].

Network pharmacology addresses the multiple key factors and targets that interact to govern associated complex pathways [6]. This concept challenges the traditional notion of treating a single disease with a single medicine that targets a single biological target. Instead, it proposes a ‟multi-component, multi-target network” and is consistent with the complexity of compositions and the involvement of multiple targets [17]. Globally, drug repurposing is becoming increasingly popular as an attractive choice due to its reduced risk, possible cost savings, and more rapid development timelines compared to developing novel drugs [18, 19]. The potential for drugs that can target numerous targets simultaneously is extremely attractive for repurposing, as this dual synergistic technique promises to improve therapeutic alternatives [20]. The conventional approach for small-molecule drug discovery emphasizes interactions between proteins and ligands. This approach is ideal for proteins such as enzymes, ion channels, or receptors since these proteins usually possess distinct binding sites for ligands, facilitating accurate interaction [21, 22].

PPIs are important for multiple biological processes and are dysregulated in complex diseases. Despite their significance, employing PPIs for therapeutic reasons has been challenging due to their complexity [23]. Modulating PPIs with small molecules was considered intricate and ‟undruggable” [24]. Due to their domain-specific and often flat attributes, small molecules pose design challenges for PPI interfaces. Inhibition was complicated due to their high-affinity binding between continuous or discontinuous amino acids and a lack of reference ligands for comparison [25, 26]. However, protein functional sites tend to aggregate within the core of their interfaces. These regions have the spatial extent of small molecules, exhibit hydrophobic characteristics, and demonstrate the ability to conform and interact with drug-like compounds dynamically. The path to successful PPI inhibitor discoveries has seamlessly blended numerous domains and utilized current approaches for targeted therapies, encompassing structural analysis, computational modeling, and biomarkers [23]. Over the last decade, cancer research has made significant advancements, particularly in studying intricate PPI targets driving the cellular processes that govern cell cycle progression, DNA repair, apoptosis evasion, and tumor suppression, such as MDM2-p53 in 2013, Bcl-2-Bax the same year, c-Myc-Max in 2014, KRAS-PDEδ in 2017, and Hsp90-Cdc37 in 2018. These accomplishments have propelled several PPI inhibitors into clinical trials, marking a promising trajectory toward novel and effective cancer therapeutics [27–31].

The study utilized the gene expression datasets from the TCGA and GEO databases to identify key biomarkers associated with LUAD. Differential gene expression analysis unveiled significant overlapping genes. The static network-based approach demonstrated a subnetwork of genes with multiple dysregulated anomalies in LUAD progression. The co-overexpression of AURKA-TPX2 was found to be associated with high survival risk in the patients, emphasizing the need for screening drugs that can inhibit the shielding potential of TPX2 in AURKA autophosphorylation and address increased cell proliferation, genomic instability, and resistance to apoptosis in LUAD [32]. Molecular docking provided a platform for repurposing 18 FDA-approved targeted cancer drugs and assessing their potential to target multiple targets. It demonstrated the inhibitory potential of FDA-targeted cancer drugs on the TPX2–AURKA interaction, aiding the experimental investigators to develop targeted therapeutic strategies and improve clinical outcomes.

## Methodology

**Fig. 1:**
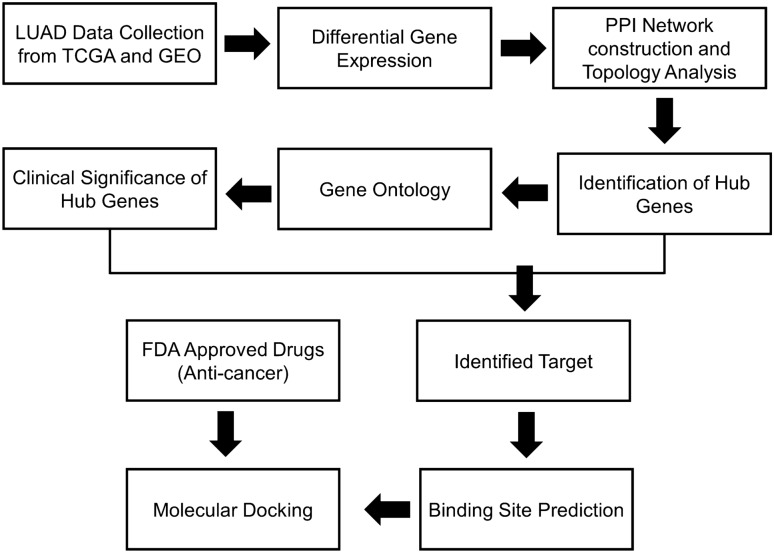
The figure illustrates an overview of the methodology implemented in this study

The study focuses on the analysis of transcriptome data from publicly accessible archives that pertain to LUAD patients. The aim was to identify clinically significant biomarkers employing a static network-based approach. FDA-approved anti-cancer drugs were repurposed for the uncovered target, revealing prospective therapeutic avenues through molecular interaction studies. Figure 1 depicts the overall workflow of the study.

### Analysis of Differential Gene Expression

The study involved the analysis of transcriptomic profiling data from TCGA-LUAD with primary tumor and adjacent tumor normal samples [33]. The analysis also included three microarray datasets, GSE10072, GSE31210, and GSE32863 (https://www.ncbi.nlm.nih.gov/geo/). Each microarray dataset had different sample sizes and platforms. The analysis focused on gene expression profiles from human LUAD tissues and adjacent normal tissues, considering DNA methylation and smoking status. The edgeR (V3.36.0) and limma (V3.50.3) packages of R (V4.1.2) were used to identify the differentially expressed genes (DEGs) in tumor vs. normal samples. DEGs were determined based on *P* value < 0.05 and log2 fold change (log2FC) > 1, with false discovery rate control using the Benjamini & Hochberg method. The overlapping DEGs were screened for determining genes differentially expressed in all four LUAD gene expression datasets using a Venn diagram (https://bioinformatics.psb.ugent.be/webtools/Venn/) [34].

### PPI Network Construction and Topological Analysis

The PPI network of the DEGs was determined using the String database (https://string-db.org/) at 5% confidence with a medium score, which aimed to exclude PPIs with low probability and enhance the reliability of the results. This approach facilitated increased coverage for a comprehensive understanding of protein interactions within the biological system, potentially encompassing less explored or transient interactions that may not be captured by high- and low-confidence networks [35]. Molecular Complex Detection (MCODE) was used to identify densely connected regions in a large PPI network. A cut-off degree of 10, cut-off node score of 0.2, K-core of 2, and a maximum depth of 100 were used as parameters. Further, CytoHubba identified the central nodes using the Maximal Clique Centrality (MCC) method [36, 37].

### Gene Ontology and Functional Enrichment Analysis

The ShinyGo (V0.77) was used for determining the Gene Ontology (GO) terms of the hub genes to describe the associated biological process, cellular component, and molecular function confined to Homo sapiens [38]. An FDR cut-off < 0.05 was used to identify statistically significant results. The use of FDR correction aided in minimizing the chances of erroneously identifying GO terms, thus enhancing the reliability and validity of the findings [39, 40].

### Survival Analysis of Hub Genes

The Kaplan–Meier (KM) plotter tool was used to compare the survival risk based on the expression of the hub genes. The genes were categorized into high- and low-expression cohorts based on the median expression values. The OS based on the LUAD data against hub genes was assessed for 200 months. For each gene, the log-rank *P* value and median survival were determined [41, 42].

### Molecular Interaction Analysis

Molecular docking of the AURKA-TPX2 complex with the 18 FDA-approved cancer-targeted therapy Drugs for in silico validation of drug in the treatment of LUAD was performed. The list of drugs was retrieved from The National Institute of Cancer (NCI) website maintained by the NIH. The AURKA-TPX2 crystal structure was downloaded from the Protein Data Bank (PDB 1OL5). AutoDock Vina was used to dock the macromolecules [43], while Adenosine diphosphate (ADP) served as control. The docking process included preparing the ligands and protein by adding hydrogen atoms, assigning Kollman charges, and removing water molecules. The active residues of the protein were determined using CASTp 3.0 and were validated with the TPX2-AURKA interaction binding pocket [44]. The docking scores of the most favorable poses of each complex were assessed [45, 46].

## Results

### Differentially Expressed Genes

**Fig. 2:**
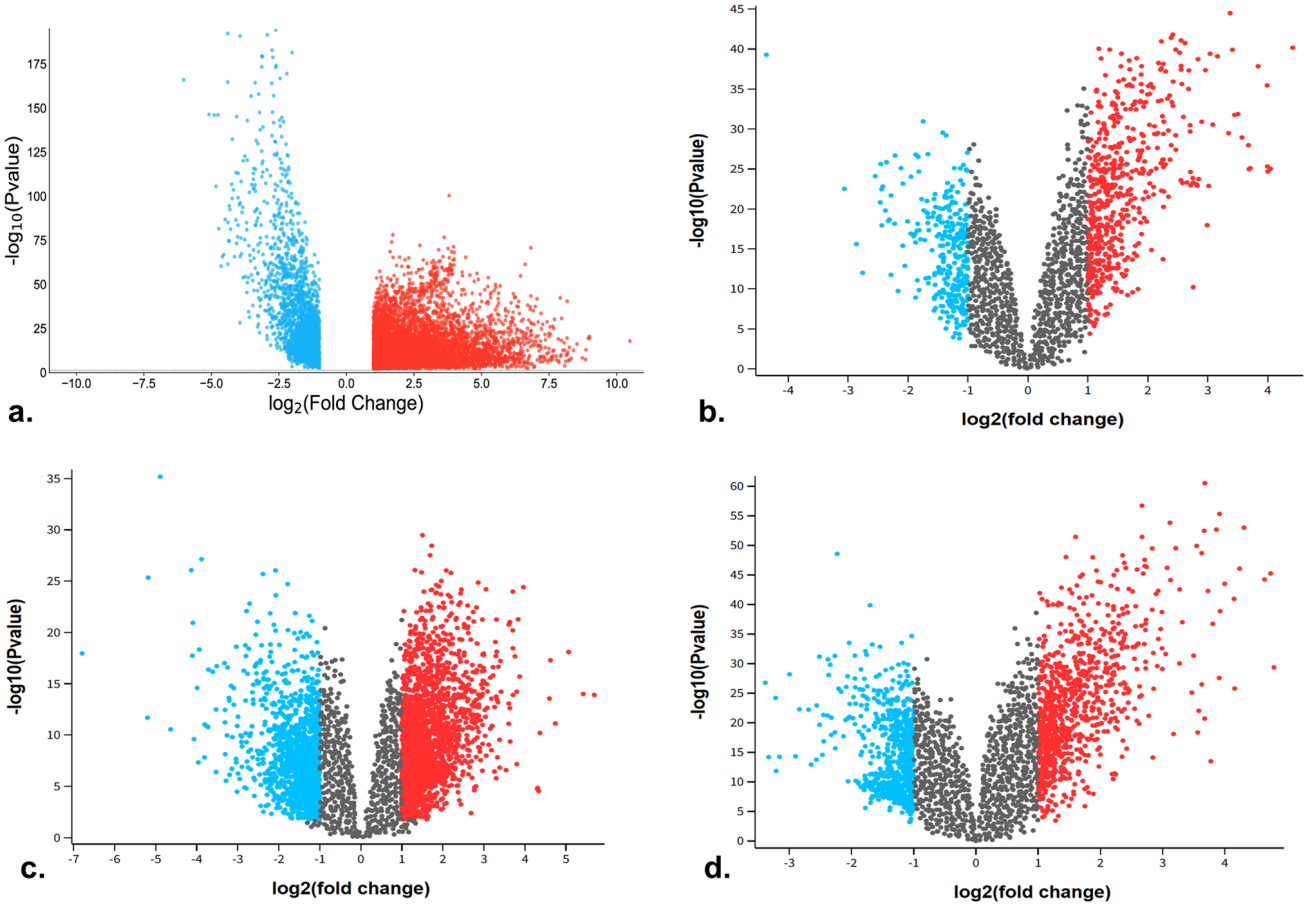
The DEGs illustrated using a volcano plot with log2FC > 1 (upregulated), log2FC <  − 1 (downregulated), and adjusted *P* value < 0.05. **a** TCGA-LUAD, **b** GSE10072, **c** GSE31210, and **d** GSE32863

**Fig. 3:**
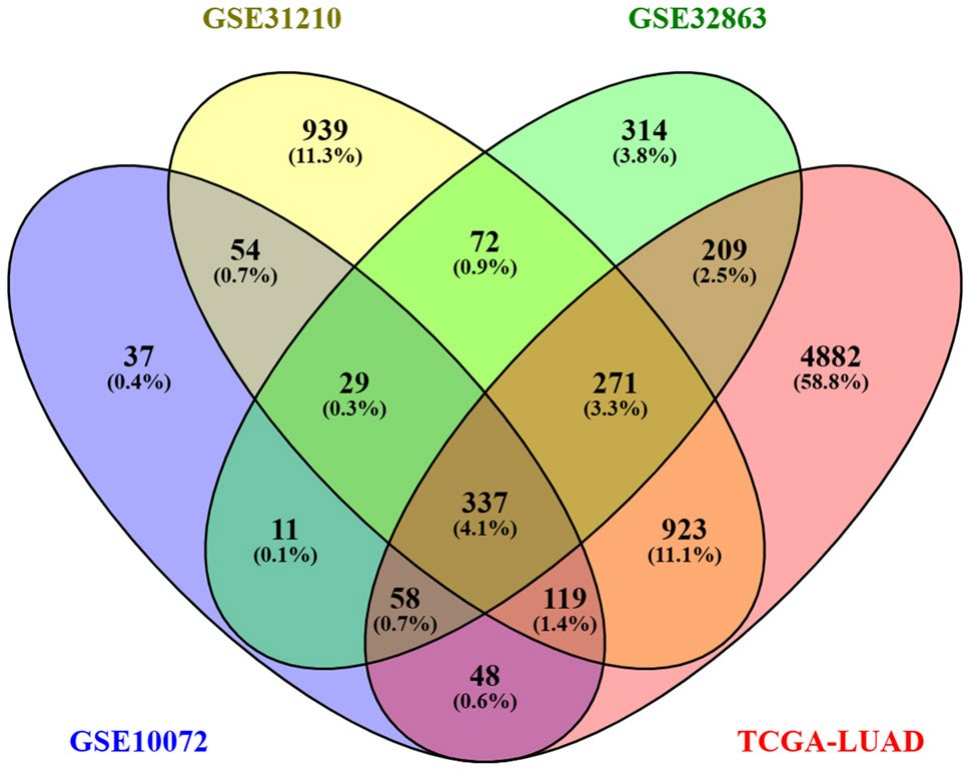
Venn diagram of the compared DEGs from LUAD expression datasets revealing the overlapping DEGs

The TCGA-LUAD transcriptomic profiling dataset comprised 537 tumor samples and 59 normal samples. Additionally, the microarray datasets GSE10072 and GSE31210, based on the GPL570 platform, consisted of 58 tumor samples and 49 normal samples, 226 tumor samples, and 20 normal samples of lung tissue, respectively. A GPL6884 expression bead chip platform-based array dataset GSE32863 contained 58 tumors and 58 normal samples that were preprocessed and analyzed with a uniform criterion of log2FC > 1 at 5% significance. The DEGs were identified and illustrated using a volcano plot (Fig. 2). The number of up- and downregulated genes is shown in Table 1. The Venn diagram revealed 337 overlapping DEGs (Fig. 3).

**Table 1:** The total number of DEGs and the number of up- and downregulated DEGs identified from the differential gene expression analysis of the LUAD expression datasets

Dataset	Total DEGs	Upregulated	Downregulated
TCGA-LUAD	6847	5166	1681
GSE10072	693	215	478
GSE31210	2744	1217	1527
GSE32863	1301	551	750

### PPI Network and Hub Genes

**Fig. 4:**
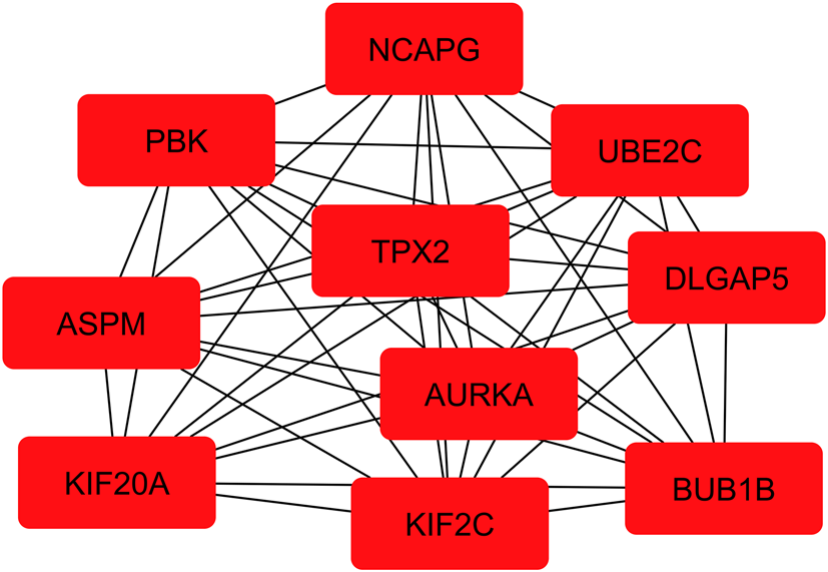
The top-ranked hub genes were identified using the MCC algorithm

The String database generated a network of significant protein-coding genes with medium confidence. As a result, a network consists of 337 nodes and 2870 edges with a clustering coefficient of 0.435 (Supp Fig. 2a). MCODE identified six clusters of densely connected nodes within the network. Cluster 1, consisting of 62 nodes and 1881 edges, achieved a score of 61.672, indicating its significance compared to the scores of the other clusters (Supp Fig. 2b). The cluster identified the top hub genes using the MCC method (Fig. 4), which outperforms other centrality algorithms in accurately assessing the importance of nodes in terms of their network structure. We observed network interactions among these nodes, which supported their potential roles as key regulators in the network.

### Gene Ontology and Functional Enrichment of Hub Genes

**Fig. 5:**
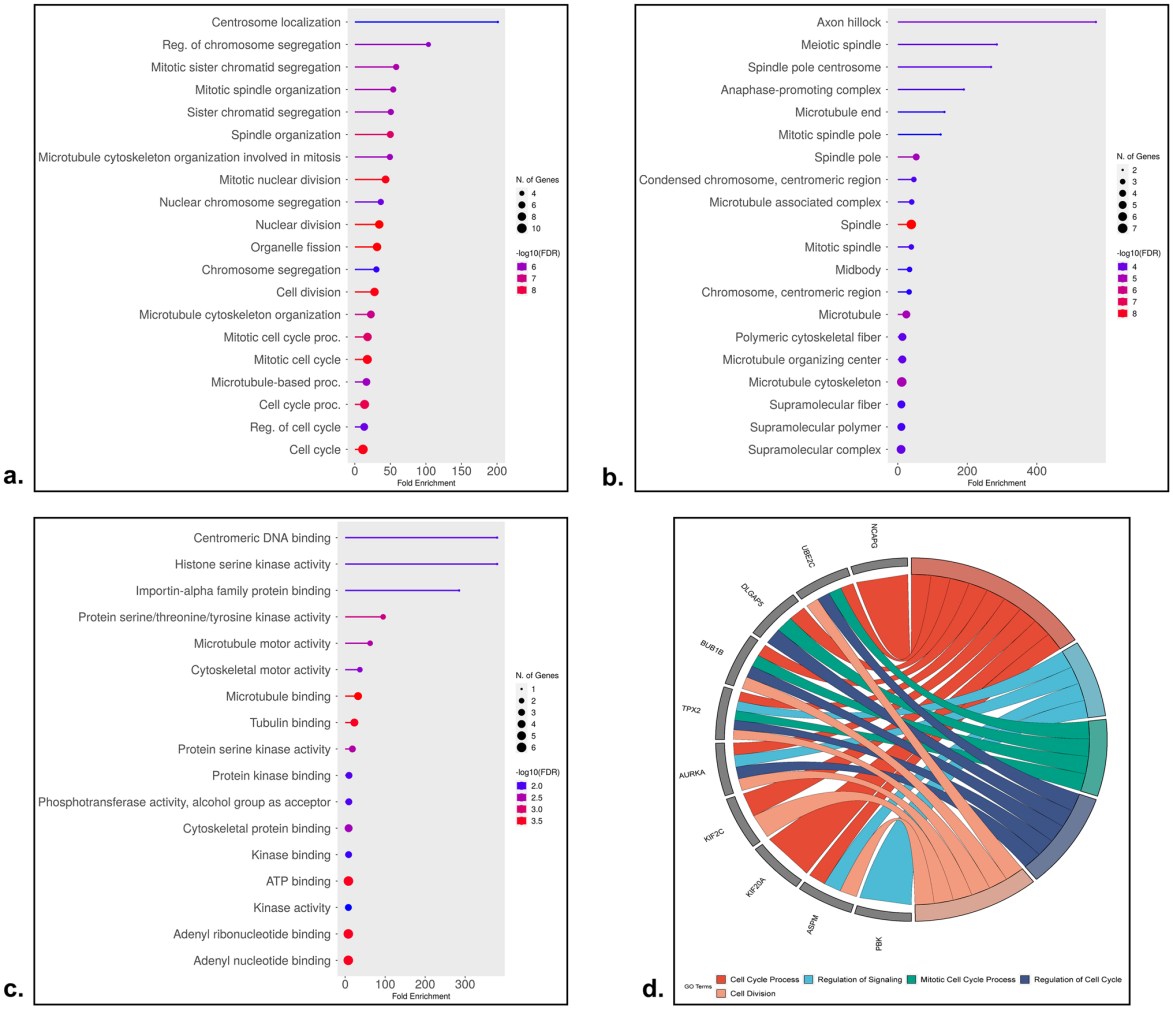
Gene functional enrichment of the hub genes based on fold enrichment at 95% confidence. **a** GO Biological process, **b** GO Cellular component, **c** GO Molecular function, and **d** the top 5 GO terms enriched to the hub genes illustrated using GO chord plot

The number of hub genes enriched to the GO terms was determined based on fold enrichment at FDR < 0.05. The GO analysis uncovered the biological process (Fig. 5a), cellular component (Fig. 5b), and molecular function (Fig. 5c). A captivating revelation emerged as we uncovered the narrative of the dysregulated hub genes in LUAD was significantly related to cell cycle regulation, mitotic cell cycle, and cell division. The cellular components, such as the spindle, microtubule, and spindle pole, took the spotlight. Furthermore, these hub genes’ molecular functions have been correlated to Adenosine triphosphate (ATP) binding, microtubule binding, and tubulin binding. The hub genes enriched to the top 5 GO terms were involved in cell cycle regulation, mitotic cell cycle processing, regulation of signaling, and cell division, which was illustrated using a GO chord diagram (Fig. 5d).

### Clinical Significance of the Hub Genes

**Fig. 6:**
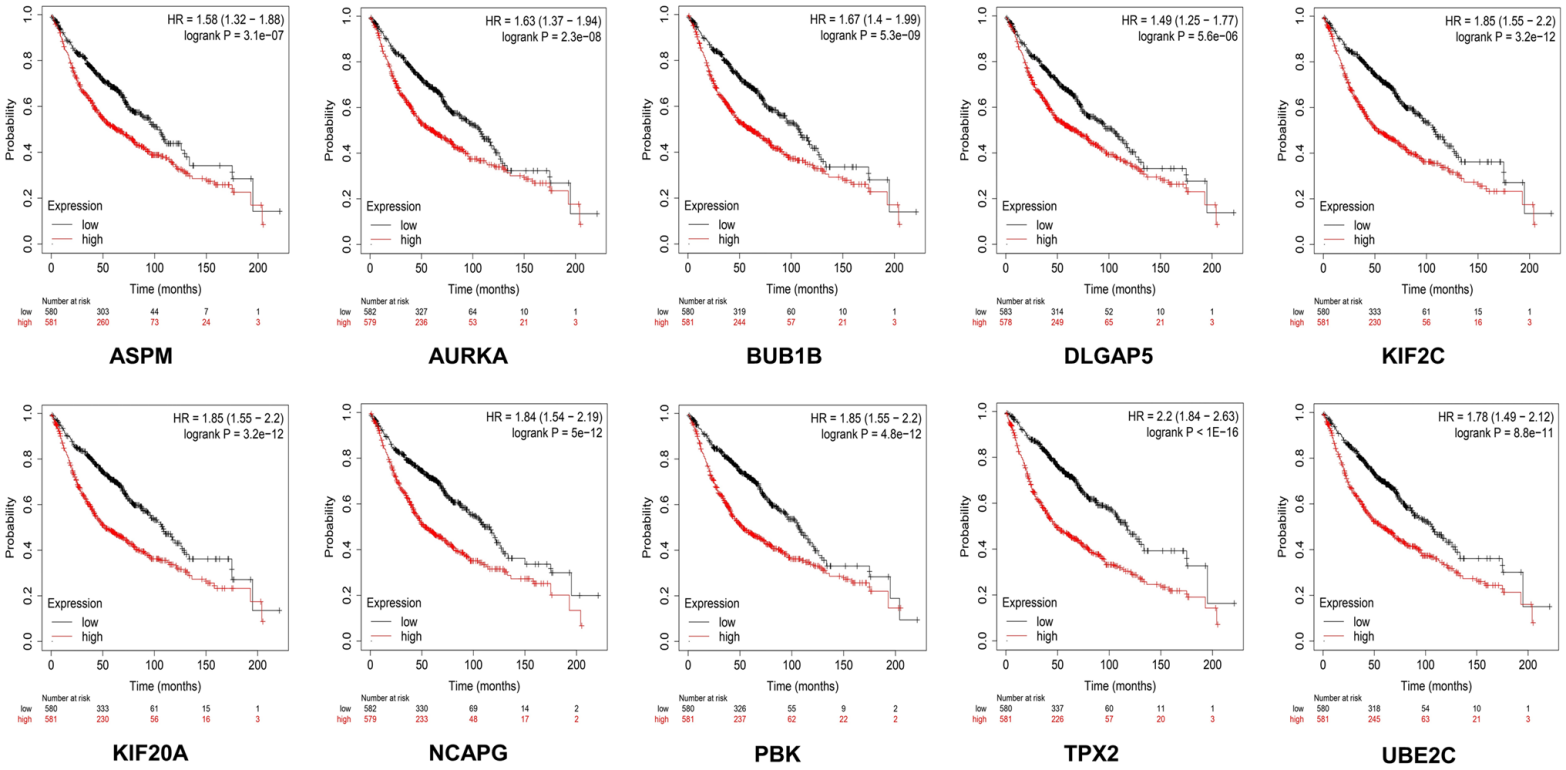
The KM plots of the hub genes represent their survival risk based on the median survival of LUAD patients for 200 months

The KM plotter assessed the expression of hub genes and their relationship with OS risk in LUAD patients for 200 months (Fig. 6). The results revealed that the overexpression of these hub genes was associated with unfavorable OS rates in patients. The hub genes showed higher hazard ratios (HR) > 1 and log-rank* P* values < 0.05. The median survival expression in months was significantly higher in the low-expression cohort compared to the high-expression cohort (Table 2). The expression pattern of the hub genes in the selected gene expression datasets is listed in (Table 3). TPX2 and AURKA possessed higher expression cohorts in all four datasets with a high survival risk in the LUAD.

**Table 2:** The median of the expression cohorts in months of the ranked hub genes demonstrates the OS of LUAD patients

Hub genes	Median of low expression in months	Median of high expression in months
ASPM	103	62
AURKA	107	61.3
BUB1B	107	61.3
DLGAP5	103	66.47
KIF2C	108	52
KIF20A	110.27	52
NCAPG	106	62
PBK	107	52
TPX2	117.33	48
UBE2C	107	57

**Table 3:** The expression patterns of the hub genes based on log2FC values in all four datasets, revealing LUAD biomarkers

Hub Genes	GSE32863	GSE31210	GSE10072	TCGA-LUAD
ASPM	1.26	− 1.51	1.48	3.87
**AURKA**	**1.47**	**1.40**	**1.14**	**2.73**
BUB1B	− 1.52	1.81	1.42	3.54
DLGAP5	− 1.71	2.08	1.31	3.81
KIF2C	− 1.33	1.77	− 1.92	3.69
KIF20A	1.09	1.53	− 1.8	3.27
NCAPG	− 1.90	1.74	− 1.72	3.72
PBK	− 1.60	1.37	1.26	3.49
**TPX2**	**1.02**	**1.84**	**1.09**	**3.77**
UBE2C	− 1.31	1.34	1.24	4.20

The bold genes exhibit comparable expression patterns

### Molecular Docking

**Fig. 7:**
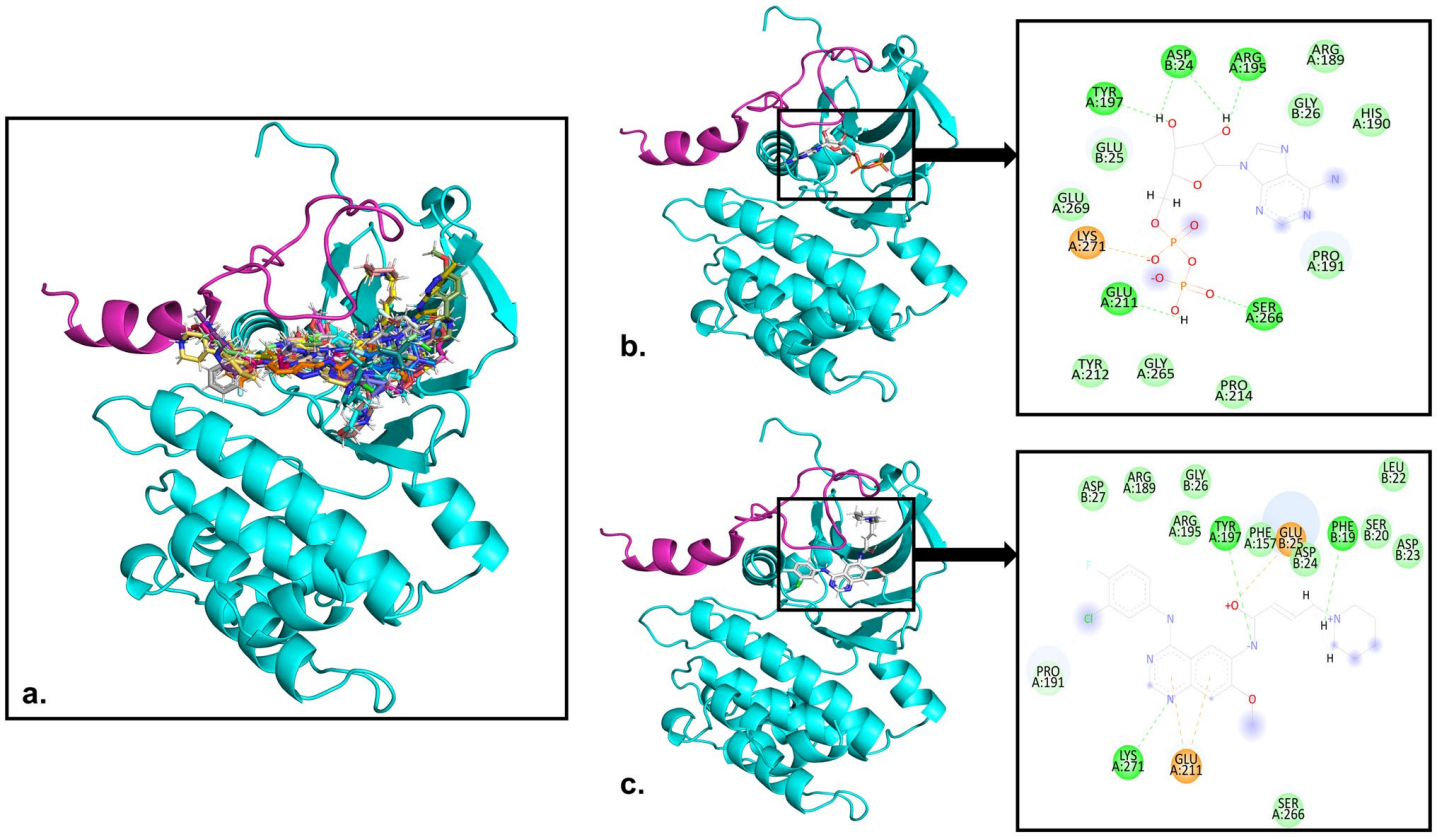
The best-docked poses of the ligands with AURKA-TPX2 complex. **a** The cancer FDA-approved drugs docked with the complex **b** ADP and **c** Dacomitinib

The analysis of molecular docking revealed that the drug molecules interacted with the binding pocket of the TPX2–AURKA interaction (Supp. Figs. 2 and 3). The complexes shown in Fig. 7 demonstrated the most favorable docking results, with a distance of < 3.5 Aº between the receptor’s binding pocket residues and the drug molecules. According to the findings, the compounds predominantly interacted with the target proteins through hydrogen bonds, electrostatic interactions, salt bridges, and hydrophobic interactions. The residues and the chains of the receptor involved in hydrogen bonding, electrostatic, and hydrophobic interactions, and the binding energies of the complexes are listed in Table 4. The binding energy of the leads to the target ranged from − 10.23 to − 5.61 kcal/mol. In contrast to ADP, Dacomitinib exhibited a higher binding affinity, establishing hydrogen bonds and negatively charged interactions and occupying the hydrophobic region of the TPX2-AURKA binding pocket.

**Table 4:** The binding energy of the FDA-approved cancer drugs with different types of interactions < 3.5 Aº with the AURKA (Chain A)-TPX2 (Chain B) complex

Compounds	Binding energy (kcal/mol)	Hydrogen bond interactions	Other interactions
ADP (control)	− 10.1	B:ASP24, A:ARG195, A:TYR197, A:GLU211, and A:SER266-conventional H-bonds A:GLU211 and A:SER266-carbon H-bonds	A:LYS276-Salt bridge
Dacomitinib	− 10.23	A:TYR197, A:LYS271, and B:PHE19-conventional H-bond B:PHE19 and B:GLU25-carbon H-bond	A:GLU211- Pi anion
Afatinib	− 9.87	A:SER266, LYS271, and PHE19-conventional H-bond B:PHE19 and A:GLU211-carbon H-bond	GLU25-Electrostatic
Brigatinib	− 9.54	A:SER266-conventional H-bond B:GLY26, B:ASP27, B:GLN29, B:ASN30, B:ASP32, A:ARG189-carbon H-bonds	B:ASP27-Salt bridge
Tepotinib	− 9.44	B:ASP27 and A:TYR212-conventional H-bonds B:ASN30, B:ASP32, and A:GLU211-carbon H-bond	B:ASP27-Salt bridge
Selpercatinib	− 8.59	B:GLY26, A:ARG195, and A:SER266-conventional H-bonds A:ILE158, A:ARG189, A:ARG195, A:LYS271, and A:GLU269-carbon H-bonds	A:GLY-Pi anion
Alectinib	− 8.04	B:ASP27, B:ASN30, B:ASP32, and A:ARG189-carbon H-bonds	B:ASP27-Electrostatic and salt bridge
Gefitinib	− 7.61	B:ASP24 and A:SER266-conventional H-bond A:PRO191, SER266, and A:GLU269-carbon H-bond	B:GLY26, A:HIS190, and A:PRO191-Halogen A:GLU211-Pi anion A:GLU269-Electrostatic and salt bridge
Entrectinib	− 7.43	B:GLU25-conventional H-bond B:ASP24, B:GLY26, and A:TYR197-carbon H-bonds	B:ASP32-Halogen
Osimertinib	− 7.39	B:GLU25, B:GLU26, B:ASP27, B:ASP32, and A:GLU211-Carbon H-bonds	B:GLU25-Salt bridge
Crizotinib	− 7.22	A:GLU269-conventional H-bond B:GLU25, B:GLY26, and A:GLU211-carbon H-bonds	B:ASP24-Halogen, A:GLU211-Pi anion, A:LYS271-Pi cation, and A:GLU269-salt bridge
Ceritinib	− 7.21	A;LYS271-Conventional H-bond B:GLY26 and B:ASN30-Carbon H-bonds	B:ASP27-Pi anion and B:ASP32-salt bridge
Dabrafenib	− 6.97	B:GLU25, A:ARG195, A:GLY211, and A:TYR212-conventional H-bonds A:ARG189, A:ARG195, and PRO214-carbon H-bonds	B:GLU25-Halogen and Pi anion A:GLU211-Pi anion
Erlotinib	− 6.63	A:GLU211 AND A:LYS271-conventional H-bonds B:ASP24. A:ILE158,and A:SER266-carbon H-bonds	A:ARG195-Pi alkyl A:GLU211-Pi anion
Pralsetinib	− 6.57	B:GLU25 and A:LYS271-conventional H-bonds A:HIS190-carbon H-bond	-
Trametinib	− 6.48	B:GLU25 and A:TYR197-Conventional H-bond B:ASP24, B:GLU25, and A:PRO191-Carbon H-bonds	A:GLU211-Pi anion GLU25-Hydrophobic
Adagrasib	− 6.15	A:SER266-Conventional H-bond A:PRO214, A:GLY265, A:SER266, and A:GLU269-Carbon H-bonds	A:GLU211-Pi anion and A:LYS271-Pi cation
Lorlatinib	− 5.98	A:TYR212 and A:LYS271-conventional H-bond A:ILE158, A:GLU211, and A:SER266	B:GLU25-Halogen and A:GLU211-Pi anion
Sotorasib	− 5.61	A:HIS190-Conventional H-bond B:GLU25-Carbon H-bond	A:GLU211-Pi anion

## Discussion

Lung adenocarcinoma is a prevalent form of lung cancer and a leading cause of cancer-related deaths [47]. As genomic and proteomic data become more accessible, accurately identifying target drugs has become increasingly important. Targeted therapies offer significant potential for effectively treating LUAD, making the identification of such therapies essential for developing successful treatment approaches [48]. Therefore, the study involved identifying therapeutic targets by analyzing the transcriptomic datasets of the primary tumor in contrast to the adjacent tumor normal samples [49].

Our study used comprehensive in silico techniques to identify genes associated with LUAD by analyzing data from the TCGA and GEO databases. Unlike most previous studies, which focused on specific genetic events or cohort analysis, we used a broader approach. As a result, we discovered 337 overlapping DEGs. The association between important DEGs regarding their physical and functional relationships was determined using a PPI network [50]. The topology analysis of the network aided in determining the significant clusters within the network, focusing on densely interconnected regions [51]. The aim was to gain insights into the critical genes, their interrelationships, and their involvement in regulating cancer-related biological processes induced by aberrant DNA methylation status and smoking status of LUAD patients. The top 10 highly ranked hub genes were identified using the MCC centrality metric with participation in the largest cliques within the network, holding significant importance and being involved in critical biological processes that contribute to cancer progression [51, 52]. Gene ontology of the hub genes provided structured terms for describing molecular functions, biological processes, and cellular components [53].

The overexpressed hub genes were predominantly enriched to cell cycle regulation and mitotic cell cycle, along with cellular components, such as the spindle and molecular functions involving microtubule binding, tubulin binding, and ATP binding, holistically triggered the critical cellular events were involved in LUAD pathogenesis [54–56]. As a result, mitotic cell cycle regulation was disrupted, resulting in uncontrolled cell growth and elevated tumor development. The chromosome segregation during cell division was impaired, leading to genomic instability due to dysregulated microtubule dynamics, influencing cell motility and intracellular transport with alterations in the energy balance for regulating a wide range of cellular processes [57–59]. This intricate combination of overexpressed hub genes endorsed mitotic errors, genetic variations, and invasive attributes, contributing to LUAD aggressiveness [60]. However, the expression patterns of the eight hub genes varied, but AURKA and TPX2 were found co-overexpressed across the datasets.

The elevated levels of AURKA hindered the tumor suppressors through phosphorylation, impeded normal functioning, and triggered the activation of oncogenic factors, resulting in chromosomal instability [61]. TPX2 is crucial in ensuring accurate assembly of the mitotic spindle. In contrast, TPX2 was closely linked to the spindle pole during mitosis. TPX2, like other mitosis-regulating proteins, was associated with unfavorable prognoses and linked to enhanced proliferation, invasion, and migration capabilities [62]. TPX2 activated AURKA by attaching it to its N-terminal domain, which shielded AURKA from dephosphorylation. Therefore, the study demonstrated the significance of targeting co-overexpressed TPX2 and AURKA could present a promising and innovative therapeutic approach [63]. Moreover, experimental and structural studies have validated the interaction between TPX2 and AURKA at the mitotic spindle [64]. The implementation of KM plots is crucial in the process of selecting biomarkers that have the potential to predict both therapeutic response and clinical outcomes [65]. The study elucidated the survival risk associated with the expression patterns of hub genes in NSCLC patients over 200 months. The hub genes showed a lower median expression in cohorts with high expression levels, indicating their involvement in impaired cell cycle regulation. This dysregulation increased the survival risk in LUAD patients, as indicated by an HR > 1, signifying a higher level of risk [66]. The KM plots indicate a higher risk of high-expression cohorts of AURKA and TPX2, which has opened an avenue for targeting the AURKA-TPX2 complex to inhibit AURKA autophosphorylation in the progression of LUAD. This implies that PPI inhibitors targeting this specific interaction could potentially overcome the specificity challenges faced by ATP-based inhibitors to some extent [67].

The study focused on determining the inhibitory potential of FDA-approved cancer drugs to overcome the need to target dysregulated AURKA-TPX2 complex in lung adenocarcinoma. TKIs have been extensively used to treat various cancers [68]. They have been developed to attenuate the enzymatic activity of mutant tyrosine kinases that contribute to the malignant traits of cells by blocking the ATP-binding sites [69]. The molecular docking study demonstrated the binding potential of the second-generation EGFR-tyrosine kinase in contrast to the ADP, which served as control. The catalytic activity of AURKA involves ATP hydrolysis to release ADP and bind to the receptor, releasing energy to facilitate autophosphorylation [70]. Dacomitinib interacted with AURKA at TYR197, LYS271, and GLU211 and TPX2 at PHE19 and GLU25 with non-covalent interactions and possessed strong binding affinity with the complex [71]. The hydrogen bond formation of dacomitinib with the receptor at TYR197 demonstrated strong evidence of exerting pharmacological actions on TPX2, shielding the dephosphorylation at the tyrosine residues during the mitotic cell cycle due to the dysregulation of protein tyrosine phosphatase [32]. The findings revealed that screened drugs occupied the hydrophobic residues of the receptor’s interaction pocket and illustrated the potential to impede the AURKA–TPX2 interaction in LUAD progression [46]. It is widely recognized in computational drug development that integrating a drug into healthcare necessitates multiple modifications and advancements [72]. These drugs could evolve as promising therapeutic agents for inhibiting the dysregulated protein–protein interactions in lung adenocarcinoma through rigorous in vitro and clinical investigations.

## Conclusions

The study determined that 337 DEGs were differentially expressed across the transcriptomic datasets of LUAD samples. The downstream analysis of the DEGs using a network-based approach determined that the densely interconnected nodes were predominantly involved in suppressing tumor suppressors, dysregulated mitotic cell cycle, and driving genomic instability due to impaired chromosomal segregation during cell division. These events were endorsed due to the co-overexpression of AURKA and TPX2 across the LUAD samples. The survival analysis of these hub genes revealed their clinical significance to be recognized as a critical therapeutic target, which has broadened the knowledge for targeting the AURKA-TPX2 complex in LUAD progression. The FDA-approved cancer-targeted drugs revealed the strong binding potential to the hydrophobic residues of the AURKA–TPX2 interaction pocket. Dacomitinib overperformed in the molecular docking studies, held with hydrogen and electrostatic interactions with both the chains and occupying the interaction pocket of the receptor. This study demonstrated an innovative targeted therapeutic strategy and addressed the knowledge gap on the pharmacological potential of FDA-approved cancer drugs in disrupting the AURKA–TPX2 interaction. Consequently, further in vitro evaluations and clinical studies of these drugs, coupled with structural modifications, would enhance drug-like properties and overcome the acquired drug resistance in LUAD patients, which holds the potential to develop a promising novel targeted therapeutic approach.

## Supplementary Information

Below is the link to the electronic supplementary material.

Supplementary file1 (DOCX 2235 KB)

Supplementary file2 (XLSX 41 KB)

## Data Availability

All authors ensure that data and materials are available and transparent.
