# Supplementary material for: Unveiling Potential Targeted Therapeutic Opportunities for Co-Overexpressed Targeting Protein for Xklp2 and Aurora-A Kinase in Lung Adenocarcinoma
Source: Mol Biotechnol. 2023 Sep 28;66(10):2792–803. doi: 10.1007/s12033-023-00879-9 (PMC11467107; doi:10.1007/s12033-023-00879-9)
Supplement: Supplementary file 1 — Supplementary file1 (DOCX 2235 KB) [file 12033_2023_879_MOESM1_ESM.docx]

**Unveiling Potential Targeted Therapeutic Opportunities for Co-Overexpressed** **Targeting Protein for Xklp2 and** **Aurora-A Kinase in Lung Adenocarcinoma**

**Supplementary Material**


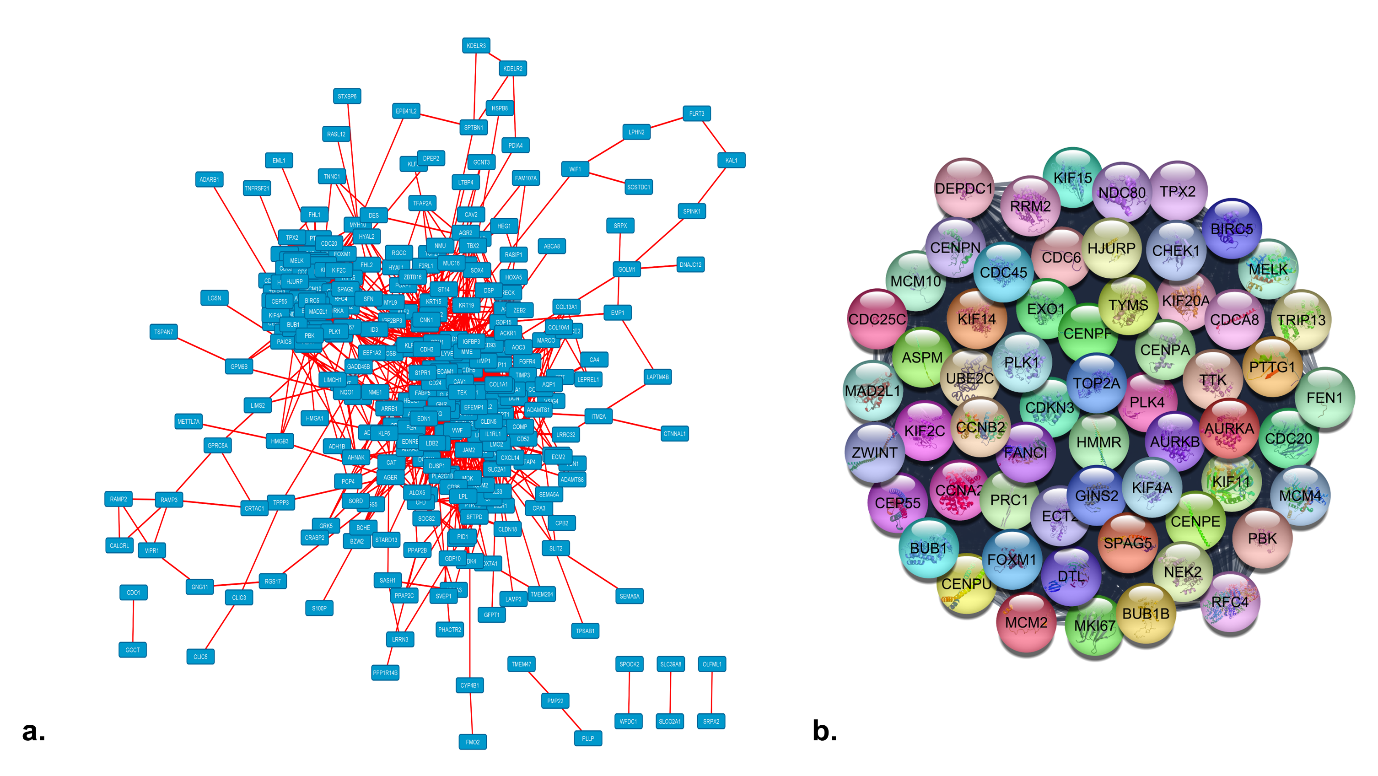


**Supp. Fig. 1.** The network construction and topological analysis. (a) PPI network generated from the DEGs. (b) A significant cluster identified from the network with a score of 61.672.


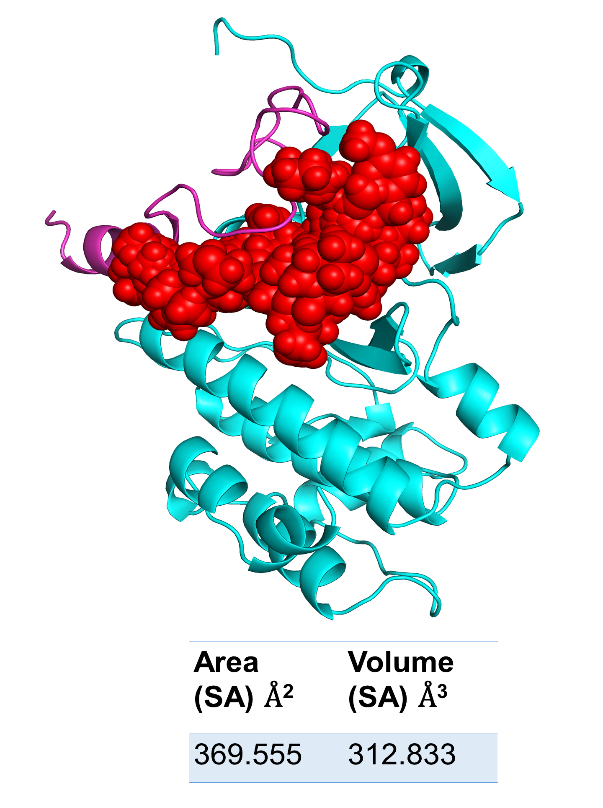


**Supp. Fig. 2.** The predicted interaction binding site of the AURKA-TPX2 complex.


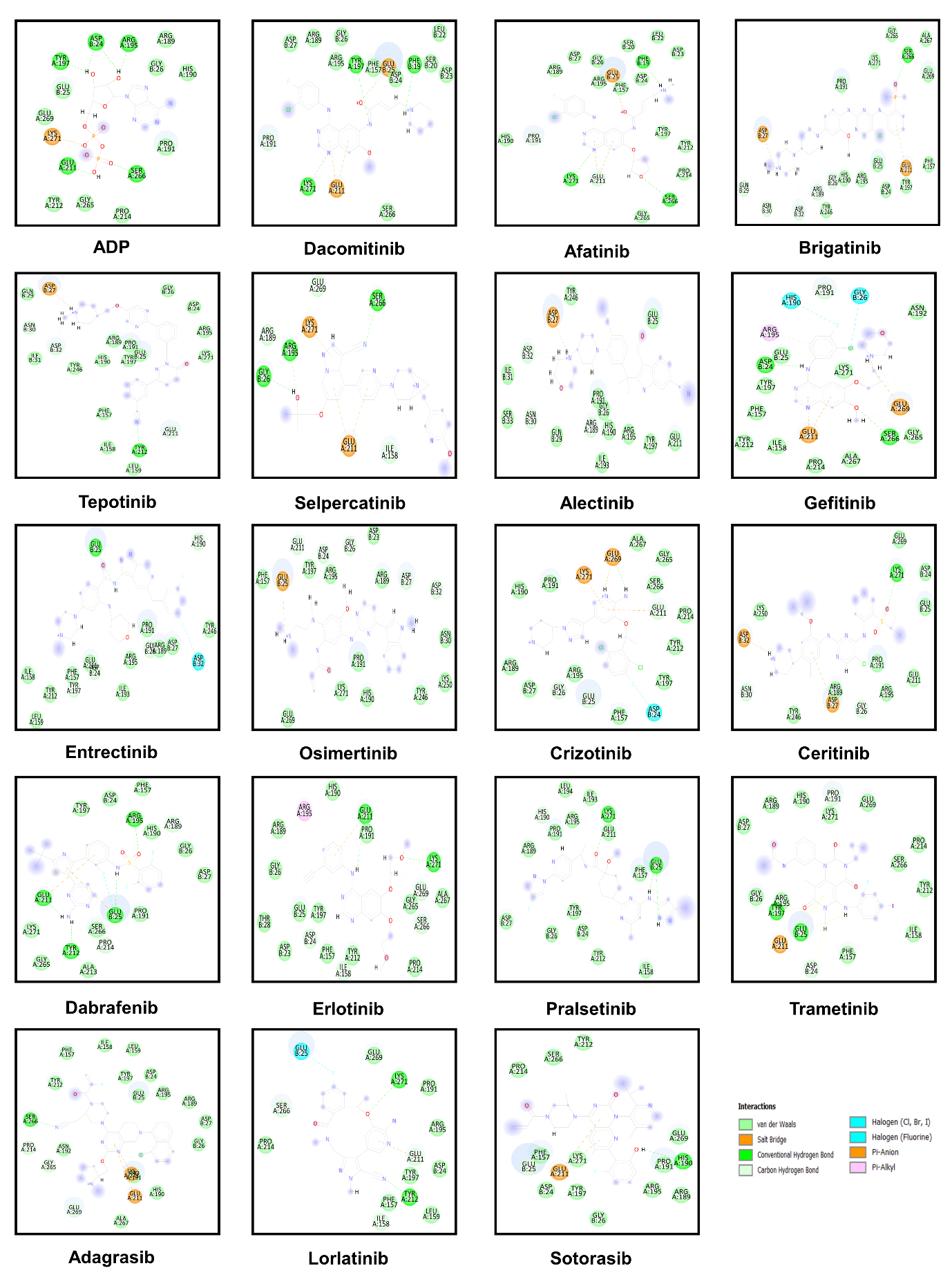


**Supp. Fig. 3.** The 2D interaction diagrams of the best-docked poses of the cancer FDA-approved drugs with AURKA-TPX2 complex.
